# Nonlinear Constrained Moving Horizon Estimation Applied to Vehicle Position Estimation

**DOI:** 10.3390/s19102276

**Published:** 2019-05-16

**Authors:** Jonathan Brembeck

**Affiliations:** Institute of System Dynamics and Control, Robotics and Mechatronics Center, German Aerospace Center (DLR), 82234 Weßling, Germany; jonathan.brembeck@dlr.de; Tel.: +49-8153-28-2472

**Keywords:** automotive applications, nonlinear observer, Kalman filter, constrained estimation, nonlinear gradient descent search, vehicle state estimation, moving horizon estimation, GNSS, IMU, INS

## Abstract

The design of high–performance state estimators for future autonomous vehicles constitutes a challenging task, because of the rising complexity and demand for operational safety. In this application, a vehicle state observer with a focus on the estimation of the quantities position, yaw angle, velocity, and yaw rate, which are necessary for a path following control for an autonomous vehicle, is discussed. The synthesis of the vehicle’s observer model is a trade-off between modelling complexity and performance. To cope with the vehicle still stand situations, the framework provides an automatic event handling functionality. Moreover, by means of an efficient root search algorithm, map-based information on the current road boundaries can be determined. An extended moving horizon state estimation algorithm enables the incorporation of delayed low bandwidth Global Navigation Satellite System (GNSS) measurements—including out of sequence measurements—as well as the possibility to limit the vehicle position change through the knowledge of the road boundaries. Finally, different moving horizon observer configurations are assessed in a comprehensive case study, which are compared to a conventional extended Kalman filter. These rely on real-world experiment data from vehicle testdrive experiments, which show very promising results for the proposed approach.

## 1. Introduction

Many demanding mechatronic systems, like the German Aerospace Center’s (DLR’s) ROMO (short for ROboMObil—DLR’s robotic electric vehicle) [1], employ state dependent nonlinear optimization-based control (e.g., [2]), which needs an accurate knowledge of the system states. Often, these states cannot be gathered directly through sensors, as an appropriate measurement principle for the searched quantity is not available (e.g., determination of the state of charge of a battery), or the sensor is expensive and therefore it is desirable to be economized (e.g., vehicle over ground velocity sensing). Especially for future autonomous vehicles, it is necessary to determine an accurate vehicle position so as to guarantee a reliable vehicle path/trajectory generation, and to follow control functionality cf. [2,3,4].

Here, a novel nonlinear position estimator, relying on a moving horizon estimator approach, which fuses inertial navigation system (INS) and Global Navigation Satellite System (GNSS) sensor data based on a model-based observer framework [2] is proposed. This framework relies on multiphysical prediction model design in Modelica [5], and an automated tool chain to incorporate these by means of the Functional Mockup Interface (FMI) [6] technology, with well proven nonlinear Kalman filter-based algorithms (cf. e.g., [2,7]).

### State of the Art in GNSS/INS Coupled Estimation

The topic of INS- and GNSS-based sensor fusion for different applications in ground or air vehicles was a large scope in the research of the last decades. A quiet comprehensive overview of methods such as dead reckoning can be found in the literature, for example, [8,9,10]. Moreover, in the literature [11], time delayed sensor fusion for sigma point Kalman filters (SPKF) is presented. In this approach, the system state is augmented with an N samples delayed state, and a cross correlation of the lagged measurement with the covariance at $t_{k-N}$ and with the covariance of the actual time instance $t_{k}$ is performed. In the literature [12], the inertial and magnetometer measurements are delayed to correspond with the GNSS data, and then fused in an observer, resulting in delayed estimates. The current estimates are afterwards calculated integrating the IMU data with the delayed estimates as the start values. Both approaches try to incorporate the delayed information of the GNSS data with a postponed prediction [12] or correction [11].

On the contrary, here, a methodology is proposed that incorporates all of the past, delayed data in every estimation step by means of a nonlinear multiphysical vehicle prediction model and a real-time capable nonlinear moving horizon estimation (MHE) algorithm. Additionally, the knowledge of the road boundaries, for example, provided by a map system with a virtual horizon, are incorporated so as to restrict the vehicle position within the carriage way. A different approach incorporating a direct collocation technique in the MHE formulation was used for the estimation of an aircraft trajectory in the literature [13]. On the contrary, in [14] a Gauss–Newton method was used to solve the nonlinear least-squares problem of the MHE formulation of a Global Positioning System (GPS) and dead reckoning integrated navigation system, by linearizing the optimization problem along the previous state estimation in each iteration. Another method to compensate the missing data of the slow sampled measurements in a (linear) MHE formulation with bounded measurement noise is proposed by the authors of [15], by means of the introduction of prediction values in the missing data gaps. The interested reader can find examples of the recent research advances in the development of new moving horizon estimation techniques in the literature [16,17].

## 2. The Observer Setup–Problem Formulation of the MHE-Based GNSS/INS Fusion

In a realistic application with a consumer quality GPS sensor, a latency between 50–1000 ms [11,12] is usually considered. This implies a drastic delay in comparison to other in-vehicle sensor systems, for example, the inertial measurement unit (IMU). Here, this fact motivates the discussed observer example with highly delayed measurements in a vehicle position estimation application.

**Figure 1:**
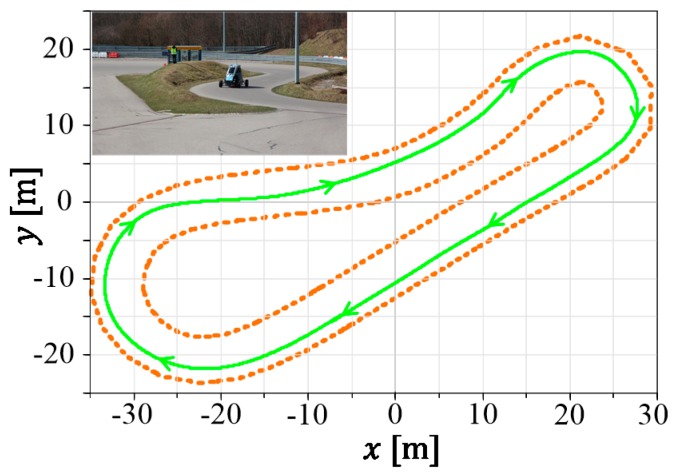
Path following control (PFC) experiment with ROMO (short for ROboMObil—German Aerospace Center’s (DLR’s) robotic electric vehicle) at the General German Automobile Club’s (ADAC’s) test facilities in Kempten.

For the experimental evaluation of the constrained state estimation of DLR’s ROboMObil, several test campaigns were carried out at the ADAC (General German Automobile Club) vehicle testing ground in Kempten, Germany. Here, tests of an advanced version of the interactive vehicle path following control (PFC) [2,3] have been performed. In the top left of Figure 1, a portrait of the testing track is shown. The green line depicts the preplanned vehicle path, while the road boundaries are represented by the dotted-orange lines.

**Figure 2:**
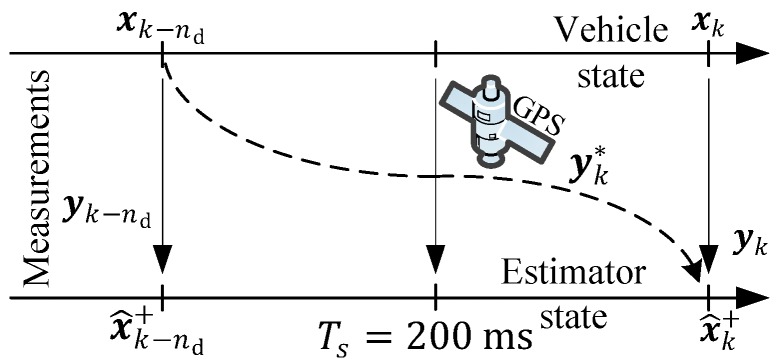
Delayed Global Positioning System (GPS) measurements incorporation in vehicle position estimation.

The ground-truth data was gathered with help of a differential GPS in order to guarantee high fidelity vehicle state measurements for the experimental validation. In Figure 2, the situation of the MHE estimator proposed here with delayed measurements is depicted. At the current time instance $t_{k}$, the estimator receives the measurements of the current vehicle yaw rate state ($y_{k}={\dot{\psi }}_{\mathrm{IMU}}^{C}$) and the delayed measurements ($y_{k}^{*}=p_{C_{\mathrm{GPS}}}^{I}=\left\{x_{c_{\mathrm{GPS}}}^{I},y_{c_{\mathrm{GPS}}}^{I}\right\}$), which are delayed for $n_{d}$ samples, and thus belong to the past vehicle state ($x_{k-n_{d}}$). Later, it will be shown that the incorporation of these delayed measurements in a conventional one-step estimation approach (i.e., a Kalman filter) leads to a poor performance or even instability.

### 2.1. The Extended Single Track Prediction Model

**Figure 3:**
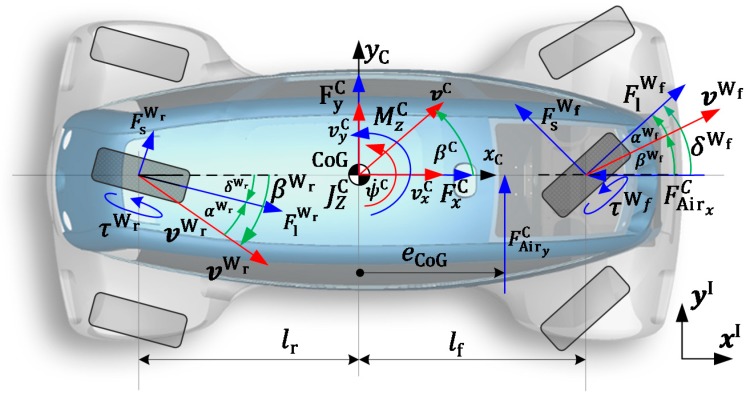
Vehicle dynamics quantities of the extended single track model.

As the scope of the proposed observer (as implied in the introduction of this section) lies on the observation of the actual vehicle states, $p_{C_{\mathrm{act}}}^{I},\psi_{C_{\mathrm{act}}}^{I},\;v_{\mathrm{act}}^{C},\;{\dot{\psi }}_{\mathrm{act}}^{C}$ for path following controllers in autonomous vehicles, as proposed by the authors of [2], an observer synthesis model reproduction depth has been chosen that meets the requirements of the computational efficiency and fidelity. An extended single track model (ESTM) was chosen for the forward driving operation mode v≥0, which incorporates vehicle standstill functionalities, rolling resistance, drag forces, and Pacejka’s Magic Formula [18] lateral characteristics of the tires. The following nomenclature is defined (cf. Figure 3):

- ${\left(\cdot \right)}^{C}$: Quantity expressed in the car coordinate system with origin in the center of gravity
- ${\left(\cdot \right)}_{C}$: Car quantity expressed in the inertial coordinate system—short for ${\left(\cdot \right)}_{C}^{I}$

The details of the extended single track model equation derivation are given in the Appendix A. It is worth mentioning that with the Modelica modeling technology, it was easily possible to integrate “if-else” constructs, which are efficiently processed by the event handling features of the compiler, while experimenting with varying discretization methods and sample rates, as the model is in continuous-time formulation. The vehicle state vector is denoted as follows, and is graphically exemplified in Figure 3:

(1) $$x^{C}=\left\{\beta^{C},v^{C},{\dot{\psi }}^{C}\;,\psi_{C},x_{C},y_{C}\right\}$$

### 2.2. Road Boundaries Constraint Formulation and Evaluation

In this section, a methodology is described for incorporating advance-known street boundaries (e.g., from digital maps in combination with vision sensors) into the vehicle position estimation. It is assumed that the initial vehicle position ($p_{C}^{I}$) is sufficiently precisely known, and the corresponding path parameter ($s^{*}$) (cf. Equation (2)) can be determined by means of a time independent path interpolation (TIPI) [2,3], which is explained in the following.

**Figure 4:**
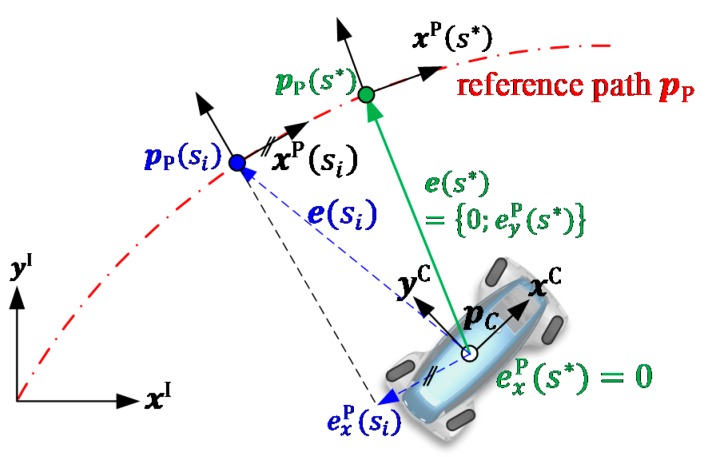
Graphical representation of the dynamic root finding to determine $s^{*}$.

Ideally, the actual path position and the position of the car coincide, $p_{P}\left(s^{*}\right)=p_{C}$ (cf. Figure 4). To approximate this condition, it is necessary to minimize the displacement (e(s)) between the vehicle and reference path, as depicted in the following:

The geometrical interpretation of this minimization objective is that $p_{P}\left(s^{*}\right)$ can be determined by projecting $p_{C}$ orthogonally on the path $p_{P}\left(s\right)$. For the TIPI this condition implies that the inverse of the maximal curvature of the demanded vehicle path defines the maximum lateral displacement for which $s^{*}$ exists: $e_{y}^{P}\left(s^{*}\right)\leq 1/\kappa_{P}\left(s^{*}\right).$

(2) $$s^{*}=\underset{s}{\mathrm{argmin}}{\left\|\underset{e\left(s\right)}{\underbrace{p_{P}\left(s\right)-p_{C}}}\right\|}_{2}$$

The interested reader can find details on how this optimization problem is solved in the literature [3].

The parametric path $\lambda_{c}\left(s\right)$ contains the following quantities for the calculation of the road boundaries constraints: the position $x_{P}^{I}\left(s\right)$ and $y_{P}^{I}\left(s\right)$ of the road middle lane, the positions of the left ($l_{x}^{I}\left(s\right),l_{y}^{I}\left(s\right))$ and the right ($r_{x}^{I}\left(s\right),r_{y}^{I}\left(s\right)$) border, and the corresponding path orientation $\psi_{P}^{I}\left(s\right)$ and its curvature $\kappa_{P}\left(s\right)$.

The extension of the vehicle prediction model (cf. Appendix A) with the roadway boundaries interpolation yields an extra state ($\dot{s}$), which is a non-physical state that belongs to the estimation task. The experimental tests considering $\dot{s}$ as an estimated state showed that this configuration leads to unsatisfactory results in the overall observer performance. Therefore, this state is separated from the state correction step in the Kalman algorithms (cf. Section 3.2).

**Figure 5:**
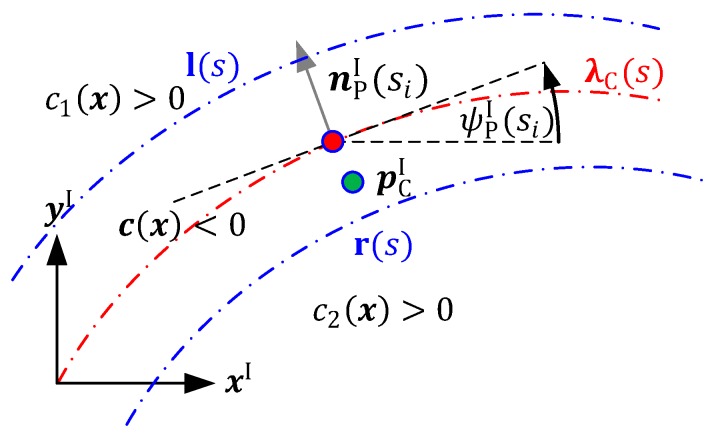
Graphical analysis of the street boundary calculation.

In Figure 5, the calculation of the roadway border constraints is graphically shown. By means of the above described algorithm, a path parameter ($s_{i}$) can be found for which the longitudinal derivation error of the vehicle’s actual position ($p_{C}^{I}$) tends to zero.

By means of the path normal vector ($n_{P}^{I}\left(s_{i}\right)$), represented in the inertial coordinate system (Equation (2)), an inequality function c(x)≤0 is calculated that penalizes the positions outside of the roadway borders, as follows:

(3) $$n_{P}^{I}\left(s_{i}\right)=\left\{-\sin\left(\psi_{P}^{I}\left(s_{i}\right)\right),\cos\left(\psi_{P}^{I}\left(s_{i}\right)\right)\right\}$$

(4) $$c\left(x\right)=\left[\begin{array}{cc} -l_{x}^{I}\left(s_{i}\right)+x_{C_{\mathrm{act}}}^{I} & -l_{y}^{I}\left(s_{i}\right)+y_{C}^{I}{}_{\mathrm{act}}\\ r_{x}^{I}\left(s_{i}\right)-x_{C_{\mathrm{act}}}^{I} & r_{y}^{I}\left(s_{i}\right)-y_{C_{\mathrm{act}}}^{I} \end{array}\right]\cdot n_{P}^{I}\left(s_{i}\right)$$

This nonlinear inequality function, c(x), can be handled directly by a posteriori constraint projection method for an (extended) Kalman filter. Details on the simplified Newton descent search algorithm used here are given in Appendix B. For the real-time capable moving horizon estimation algorithm in Section 3. it is necessary to linearize c(x) at all of the time instances $t_{k}$, where it is likely that a constraint may be violated by the estimator $c\left(x_{k}\right)>-\epsilon$, as follows:

(5) $$c\left(x\right)≅c\left(x_{k}\right)+{\frac{\partial c}{\partial x}|}_{x_{k}}\cdot \left(x-x_{k}\right)$$

By rearranging Equation (4), the linearized inequality description in Equation (5) can be formulated as follows:

(6) $$C_{k}\cdot x\leq d_{k}\;\overset{\mathrm{yields}}{\to }\underset{C_{k}}{\underbrace{{\frac{\partial c}{\partial x}|}_{x_{k}}}}\cdot x\leq \underset{d_{k}}{\underbrace{{\frac{\partial c}{\partial x}|}_{x_{k}}\cdot x_{k}-c\left(x_{k}\right)}}$$

**Figure 6:**
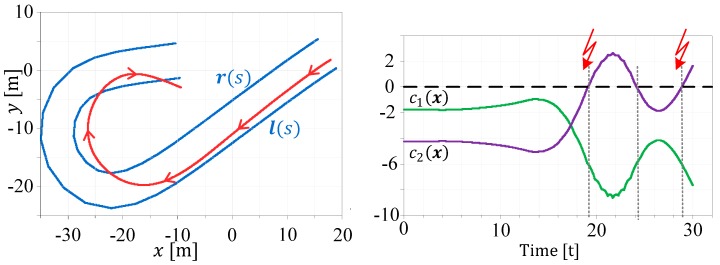
Example path with boundary violation (**Left**) and constraints evaluation (**Right**).

Figure 6 shows an example for the calculation of the nonlinear constraint function. In the left plot, a street is marked with the left l(s) and the right r(s) street boundaries, while the car (red line with direction arrows) crosses the right boundary in the hairpin curve. This leads to a violation of the right border constraint condition $c_{1}\left(x\right)>0,\;\forall \;t\in \left[19.6\;22.1\right]\;\wedge$ [29.4 30], as can be seen in the right plot of Figure 6.

**Figure 7:**
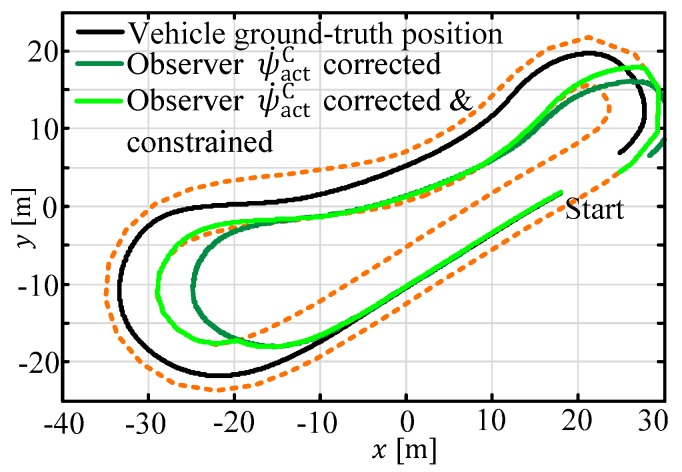
Observer behavior with and without path constraints.

Assuming that only the vehicle yaw rate (${\dot{\psi }}_{\mathrm{act}}^{C}$) is available to the (extended) Kalman filter, the position estimate would drift away, as shown in Figure 7 dark green line). Making use of the proposed boundary estimation approach used here, in combination with the inequality handling feature from the author of [2], yields a bounded and valid result (light green line), as follows:

## 3. Real-Time Nonlinear Moving Horizon Estimation

In this section, a nonlinear moving horizon estimator (MHE), which utilizes multiple past measurements to estimate the state at the current time instance, is introduced. In addition to the state constraints, delayed measurements can be directly incorporated into the problem formulation.

Referring to the literature [19], the approach of the moving horizon estimation is the reformulation of the general optimization objective of the Kalman filter theory, also known as the full-information filter. In the nonlinear case, the minimization problem can be written as follows:

(7) $$\begin{array}{l} \underset{\xi_{k}}{\min}∥x_{0}-{\hat{x}}_{0}{∥}_{I_{0}^{+}}^{2}+\overset{N}{\underset{i=1}{\sum }}∥y_{i}^{m}-h\left(x_{i}\right){∥}_{R^{-1}}^{2}+\overset{N-1}{\underset{i=0}{\sum }}∥x_{i+1}-f\left(x_{i},u_{i}\right){∥}_{Q^{-1}}^{2}\\ \xi_{k}={\left(x_{0}^{T},\;x_{1}^{T},\ldots ,x_{N}^{T}\right)}^{T} \end{array}$$

where in the nomenclature of $\underset{x}{\min}\|e{\left(x\right)\|}_{W}$ is denoted a with W, and is a weighted least squares minimization problem of a function e with respect to x. The functions f and h are the nonlinear state functions, resprectivley, of the model output equations. The matrix $I_{0}^{+}={\left(P_{0}^{+}\right)}^{-1}$ and the vector ${\hat{x}}_{0}$ represent the initial guess values of the covariance and the system state. The matrix Q denotes the covariance of the system states, and its entries constitute the confidence in the underlying prediction model and can be tuned by the application engineer. The second tuning matrix R represents the confidence in the actual measurements. The well-known Kalman filter algorithms (e.g., extended Kalman filter (EKF) and unscented Kalman filter (UKF)) are special cases of this optimization objective, in the case that only the measurements at the current time instance ($t_{k}$) are available [19]. As the dimension, N, of the optimization problem would grow tremendously with proceeding time, in MHE theory, the time span is limited to a predefined length of previous time instances and is shifted in every sample step.

**Figure 8:**
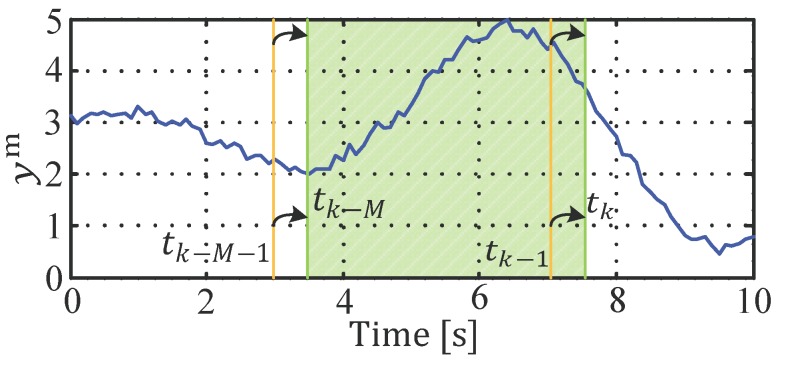
Schematic representation of a moving measurement window.

Therefore, a sliding window with M steps back from the actual time instance ($t_{k}$), is considered to smooth the state estimation. In every sample step ($T_{s}$), this window is shifted one-step ahead—in Figure 8 this is illustrated for one measurement variable, $y^{m}$. At the filter initialization ($t_{0}$), only one measurement is available, and therefore the measurement storage must be filled with r<M steps before the window starts to move. Afterwards, the window length is kept constant and all of the past M measurements are taken into account (the window with M measurements is colored in green in the subsequent figures). In other words, MHE can be seen as a real-time calculable approximation of the full-information filter.

In this way, the estimate gets more robust against external disturbances, delayed measurements can be incorporated (cf. Section 3.2.3), and also constraints can be imposed directly [19].

By means of the proposed estimation framework presented in [2], it is also possible to incorporate complex nonlinear Modelica-based prediction models to moving the horizon estimation. In the context of toolchains for observer code generation and nonlinear constraint MHE, different research studies were recently published that utilize the ACADO toolbox for embedded [20] and real-time moving horizon estimation [21,22]. They make use of the real-time iteration (RTI) scheme transferred to the MHE approach in he literature [23]. The basic strategy is to discretize the estimation problem with a multiple shooting discretization using numerical integration. Then, the main idea of the RTI scheme is to use the shifted state variables of the previous optimization run as the new linearization point, and to perform only one SQP step per sample time [20].

In this work, the problem formulation in Equation (7) is extended for nonlinear systems incorporating linear state constraints, tailored to meet real-time application restrictions by means of a nonlinear gradient descent search. Details on the reasons for this approach are given in the following section.

(8) $$\begin{array}{cc} \underset{\xi_{k}}{\min}\;g(\xi_{k} & ={\left(x_{k-M}^{T},\;x_{k-M+1}^{T},\ldots ,x_{k}^{T}\right)}^{T})\\ s.t.A\cdot \xi_{k} & =b\\ C\cdot \xi_{k} & \leq d \end{array}$$

(9) $$g\left(\xi_{k}\right)=∥x_{k-M}-{\hat{x}}_{k-M}^{+}{∥}_{I_{k-M}^{+}}^{2}+\overset{k}{\underset{i=k-M}{\sum }}∥y_{i}^{m}-h\left(x_{i}\right){∥}_{R^{-1}}^{2}+\overset{k}{\underset{i=k-M+1}{\sum }}∥x_{i}-x_{\mathrm{int},i}{∥}_{Q^{-1}}^{2}$$

(10) $$\begin{array}{c} x_{\mathrm{int},k-M}={\hat{x}}_{k-M}^{+}\\ \;x_{\mathrm{int},i}=f_{i|i-1}\left(x_{\mathrm{int},i-1},u_{i-1}\right)\\ \left(i=k-M+1,\ldots ,k\right) \end{array}$$

All of the system function evaluations were done by the FMI [6] (cf. also Section 3.2) and are marked in red in the following section. The optimization vector ($\xi_{k}$) is assembled with M subsequent discrete state vectors within the current estimation window. The optimization cost function $g\left(\xi_{k}\right)$ in Equation (9) is composed of the following three parts.

The first argument ${\left\|\cdot \right\|}_{I_{k-M}^{+}}$ is the arrival cost, which summarizes all of the available information prior to the estimation window; this can also be seen as a regulation term on the states at $t_{k-M}$ [21]. It is introduced so as to guarantee that the oldest estimation ($x_{k-M}$) is coincident with the corrected Kalman filter state estimation (${\hat{x}}_{k-M}^{+}$) weighted with the information matrix ($I_{k-M}^{+}$). Note that in every moving horizon estimation step, a Kalman filter step from k−M−1 to k−M is performed to fulfill the Kalman state estimation theory—Equation (7). The information matrix, $I_{k-M}^{+}$, is calculated via the inverse of the covariance matrix ${\left(P_{k-M}^{+}\right)}^{-1}$. As direct matrix inversion should be avoided, because of the numerical stability and accuracy, the author proposes using a square root (SR)-UKF or SR-EKF Kalman filter algorithm that uses square-root decomposition and rank 1 updates to propagate the covariance matrix in a lower triangular form, P=L⋅U. The inverse of the lower triangular (L) can be efficiently calculated by the LAPACK routine DTRTRI, and therefore, the propagated information matrix results in $I_{k-M}^{+}=L^{-1}\cdot L$.

The second argument, ${\left\|\cdot \right\|}_{R^{-1}}$ is the, over the time instances (k−M to M) summarized and with $R^{-1}$ weighted, difference between the available measurements $y_{i}^{m}$ and the output equations of the underlying prediction model $h\left(x_{i}\right)$ as function of the states of the current optimization vector $\xi_{k}$.

Finally, the third argument, ${\left\|\cdot \right\|}_{Q^{-1}}$, denotes the, over the time instances (k−M to M) summarized and with $Q^{-1}$ weighted, difference between the optimized states $x_{i}$ and the open loop integrated prediction model states $x_{\mathrm{int}}$ in Equation (10). $x_{\mathrm{int}}$ is calculated only once per optimization step by a simulation using ${\hat{x}}_{k-M}^{+}$ as start vector and $u_{i}$ as input vector.

**Figure 9:**
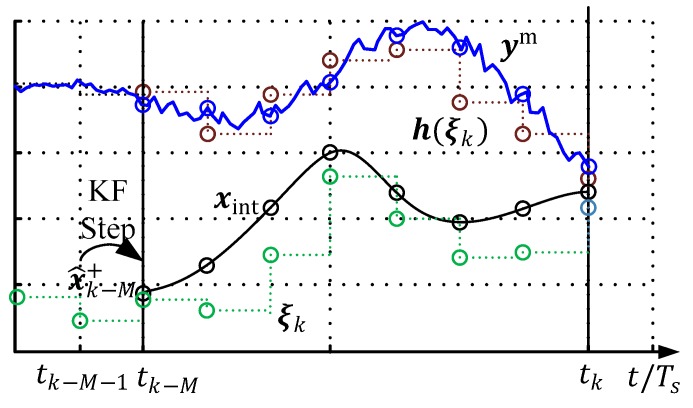
Schematic representation of a moving measurement window.

In Figure 9, a qualitative graphical interpretation of the optimization problem for a scalar problem with $n_{x}=n_{y}=1$ is shown. The circle points of the quantities denote the particular values at a discrete-time instance, which is evaluated in the objective function Equation (9).

The complete algorithm is summarized in Algorithm 1. In the first step, all of the past measurements are stored in a first in first out (FIFO) ring buffer. As long as not enough measurements for the complete window M are available, the measurements and the model inputs are appended to the buffer.
**Algorithm 1.** Algorithm for a nonlinear MHESet k=0
$\left(k\in {\mathbb{N}}^{+}\right)$ and set $x_{k}=x_{0}$
**while** (brake==false) **do**    *Fill ring buffer with measurements and system inputs:*  **if**
k<M
**then**    append $u_{k}$ to u and $y_{k}^{m}$ to $y^{m}$
  **else**    left shift one entry of u and $y^{m}$
    and append $u_{k}$ respectively $y_{k}^{m}$
  **end if**    *Optimize over stored measurement window (Equation (8)):*  **if**
k>M
**then**    *Propagate*
${\hat{x}}_{k-M-1}^{+}$ via *a Kalman Filter step (e.g., EKF or UKF* cf. 2*):*    *xKF(*${\hat{x}}_{k-M-1}^{+})\to {\hat{x}}_{k-M}^{+},\;\;I_{k-M}^{+}$    *Project states on the constrained area (c.f. Appendix B)*    $\underset{x}{\min}\|x-{\hat{x}}_{k-M}^{+}\|\;\;\;s.t.\;\;c\left(x\right)\leq 0$**end if**k=k+1**end while**

### 3.1. A Nonlinear Gradient Descent Opimization Algorithm for MHE

For the solution of the proposed MHE problem formulation, Equation (8) to Equation (10), a nonlinear gradient descent search algorithm (NG) was chosen, because for this solver, only the first derivatives of the minimization objective (Equations (8)–(10)) are needed, which is an important constraint for the available interfaces of the extended FMI 2.0 co-simulation interface (see [2]—Section 4.2.2). Furthermore, linear equality and inequality constraints can be incorporated easily, and the method can be stopped after every optimization step, still offering a reliable sub-optimal solution. The latter is important if the optimization is not finalized at the next sample instant. The algorithm of the constrained NG is given in Algorithm 2, for more details see, for example [24]. This gradient descent search algorithm is already successfully being used on a rapid-prototyping real-time system. For example, it was employed—similar to the one proposed in this manuscript—in previous research on path planning tasks [4]. This gradient descent optimization has been implemented and successfully tested on a dSpace real-time control system with a sample time of 100 ms. The gradient descent algorithm has a fixed number of maximum iterations so as to guarantee the real-time constraint. Even if the global optimum is not found, the suboptimal solution will be better than the initial start solution. Here, the implemented NG algorithm for the observer framework is tailored for the nonlinear moving horizon estimator to meet the maximum flexibility in the calculation of the gradient, and the objective function calculation. That is, it is possible for the user to modify the calculation in Modelica with replaceable function pointers, without modifying the optimization algorithm itself. This will be shown later in an example in Section 3.2.
**Algorithm 2.** Nonlinear gradient search algorithm for MHESet j=0
$\left(j\in {\mathbb{N}}^{+}\right)$ and $\xi_{k}^{0}={\left(x_{\mathrm{int},k-M}^{T},\ldots ,x_{\mathrm{int},k}^{T}\right)}^{T}$
**while**$\left|g\left(\xi_{k}^{j}\right)-g\left(\xi_{k}^{j+1}\right)\right|>10\cdot \epsilon$**do**  **if** unconstrained **then**    $r_{j}=-\nabla g\left(\xi_{k}^{j}\right)$  **elseif** constrained **then**    $r_{\mathrm{pro},j}=-P^{†}\left(A\right)\cdot \nabla g\left(\xi_{k}^{j}\right)$    $r_{f,j}=r_{\mathrm{pro},j}-\left(G_{\mathrm{pa},i}r_{\mathrm{pro},j}\right)G_{\mathrm{pa},i}^{T}$
  **end if**  *Determine step size via line search:*
$\eta_{j}=\underset{0\leq \eta }{\mathrm{argmin}}\;g\left(\xi_{k}^{j}+\eta \cdot r_{j}\right)$
  *Optimization step:*
$\xi_{k}^{j+1}=\xi_{k}^{j}+\eta_{j}r_{j}$  j=j+1
**end while**

In step 1 (line 1), an initial solution, $\xi_{k}^{0}$, is needed. Well-proven strategies for its calculation are an open loop integration of the prediction model from $x_{k-M}$ to $x_{k}$, or a left shift of the last optimization vector $\xi_{k-1}$ and appending an open loop integration from $x_{k-1}$ to $x_{k}$. In the unconstrained (lines 3–4) case of the second step, the gradient $\nabla g\left(\xi_{k}^{j}\right)$ of the descent direction can be directly calculated, as shown in Algorithm 3.
**Algorithm 3.** MHE calculation by means of functional mockup unit (FMU) evaluationsDefine: $R^{*}={\left(R\cdot R^{T}\right)}^{-1}$ and $Q^{*}={\left(Q\cdot Q^{T}\right)}^{-1}$
$\nabla g_{1:n}=\left(I_{k-M}^{+}+{\left(I_{k-M}^{+}\right)}^{T}\right)\left(x_{k-M}-{\hat{x}}_{k-M}^{+}\right)\;-2{\frac{\partial h\left(x_{k-M}\right)}{\partial x_{k-M}}}^{T}R^{*}\left(y_{k-M}^{m}-h\left(x_{k-M}\right)\right)$**for**i=k−M+1 to k**do**$\;\nabla g_{1+\left(i-k+M\right)\cdot n:\left(i-k+M+1\right)\cdot n}=\frac{\partial g\left(\xi_{k}\right)}{\partial x_{i}}=2Q^{*}\left(x_{i}-x_{\mathrm{int},i}\right)-2\frac{\partial h{\left(x_{i}\right)}^{T}}{\partial x_{i}}R^{*}\left(y_{i}^{m}-h\left(x_{i}\right)\right)$**end for**

Also, if equality constraints (lines 5–7) should be incorporated, A⋅ξ=b, the descent direction must be projected on these by means of the Moore–Penrose pseudoinverse [25] $r_{\mathrm{pro},j}=P^{†}\left(A\right)\cdot \nabla g\left(\xi_{k}^{j}\right)$. In most of the MHE applications, this step can be neglected (i.e., $P^{†}\left(A\right)=I$), as only inequality constraints are of mayor interest in order to limit the boundaries of the states. To guarantee that the descent direction does not violate the inequality constraints, a set of possible active constraints, $G_{\mathrm{pa},i}$, in the null space of the linear constraints must be determined. The descent direction is projected on the active constraints $\left(G_{\mathrm{pa},i}r_{\mathrm{pro},j}\right)G_{\mathrm{pa},i}^{T}$. More algorithm implementation details can be found in the literature [24].

In step 3 (line 9), an iterative free line search via quadratic approximation by a second order Taylor series polynomial is performed (Equation (11)). Unfortunately, the derivatives of $g\left(\xi_{k}^{j}\right)$ must be calculated via numerical differences, as the extended FMU 2.0 co-simulation interface only supports directional derivatives with respect to the system states and inputs (see [6]—Section 2.1.9). Note that the necessary determination of $g\left(\xi_{k}^{j}+\Delta s\right)$ in the differential quotient causes only evaluations of the algebraic output equations of the FMI (cf. Equation (9)), as the calculation of the initial guess $x_{\mathrm{int},i}$ is only performed once in this approach.

(11) $$\begin{array}{cc} g\left(\xi_{k}^{j}+\eta \cdot r_{j}\right) & \approx G\left(\eta \right)≔g\left(\xi_{k}^{j}\right)+g^{\prime }\left(\xi_{k}^{j}\right)\eta +\frac{1}{2}g^{\prime \prime }\left(\xi_{k}^{j}\right)\eta^{2}\\ \frac{\partial G\left(\eta \right)}{\partial \eta } & \overset{!}{=}0=g^{\prime }\left(\xi_{k}^{j}\right)+g^{\prime \prime }\left(\xi_{k}^{j}\right)\eta ,\\ \Rightarrow \eta_{j} & =-\frac{g^{\prime }\left(\xi_{k}^{j}\right)}{g^{\prime \prime }\left(\xi_{k}^{j}\right)} \end{array}$$

In step 4 (line 10), the actual optimization step, $\xi_{k}^{j+1}$, is computed. Finally, in the last step, it is checked whether the stop criterion has been reached. This can be, on the one hand, with a criterion that controls whether the change in the last iteration, j, is small $\left|g\left(\xi_{k}^{j}\right)-g\left(\xi_{k}^{j+1}\right)\right|<10\cdot \epsilon$, and, on the other hand, a real-time constraint that stops the search in order to guarantee the cycle-time of the real-time system. In this case, it is assumed that the initial guess ($\xi_{k}^{0}$), calculated by the high fidelity Modelica model, has been already a valid suboptimal solution, and the iterations of the NG did further improve it (cf. Algorithm 2), before the stop ($\xi_{k}^{j}$).

### 3.2. Moving Horizon Estimation Algorithm Extensions

Here, for the analyzed ESTM MHE observer, different extensions are discussed, in comparison to the nominal MHE algorithm formulation in Section 3. First, a computationally reliable method is introduced for the calculation of the prediction model and the observer constraints, by means of two multi-rate extended FMUs 2.0 for co-simulation (cf. [2,6,26]). It enables enlarging the sampling time of the observer, inspite of the fact that the constraint calculation needs to be executed in a 50 times faster sampling rate, which gives large benefits to the real-time capability. Second, advanced methods for the coupling of discrete optimization variables are proposed. These enforce the physical coupling of the discrete tuner states ($\xi_{k}$) to overcome an unrealistic solution of the state trajectory. Third, a heuristic method is discussed to prevent optimization freezing through intelligent recalculation of the reference trajectory ($x_{\mathrm{int}}$) in segments where no measurements are available.

#### 3.2.1. Constraint Evaluation with a Multi-Rate FMU Model Splitting Concept

**Figure 10:**
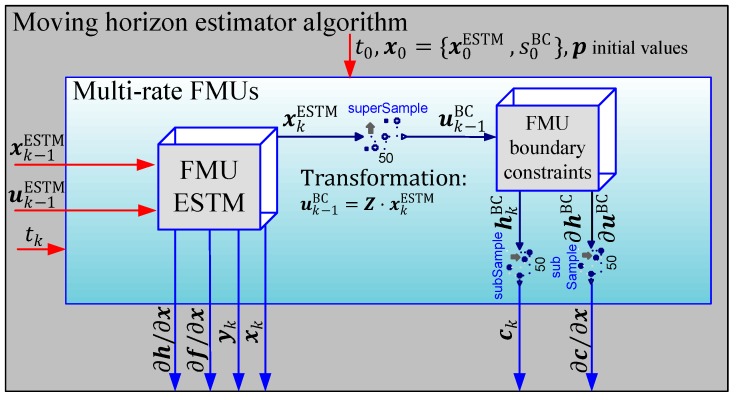
Two encapsulated multi-rate functional mockup units (FMUs) as one prediction model.

The first implementations of the constrained observer were based on a single prediction FMU that combined the ESTM prediction model (Section 3) and the boundary constraint evaluation (Section 2.2). To separate the estimated states from the state of the constraint evaluation (the path parameter s), Modelica’s logical vector indexing feature was used. However, a simulation experiment analysis showed that in this configuration, it is necessary to run the whole estimator with a fast sampling rate of $T_{s}=4\;\mathrm{ms}$. This is due to the fast dynamics of the control loop to determine the current path parameter (s) in the constraint calculation module (cf. Section 2.2). To overcome this issue, the ESTM and the boundary constraint (BC) model were split into two separate FMUs with different sample times ($T_{s}^{\mathrm{ESTM}}=\;200\;\mathrm{ms},\;\;T_{s}^{\mathrm{BC}}=4\;\mathrm{ms}$). The connection of the multi-rate FMUs and the estimation algorithm are sketched in Figure 10. Through the model splitting, the states of the ESTM FMU ($x=\left\{\beta^{C},v^{C},{\dot{\psi }}^{C},\psi_{C},x_{C},y_{C}\right\})$ are the inputs of the boundary constraints of FMU, which only has one state, the corresponding path parameter s. The sample time of the constrained FMU is 50 times higher than the one of the ESTM, so as to guarantee numerical stability. Z denotes the permutation matrix between the inputs of the BC FMU and the states of the ESTM FMU.

**Figure 11:**
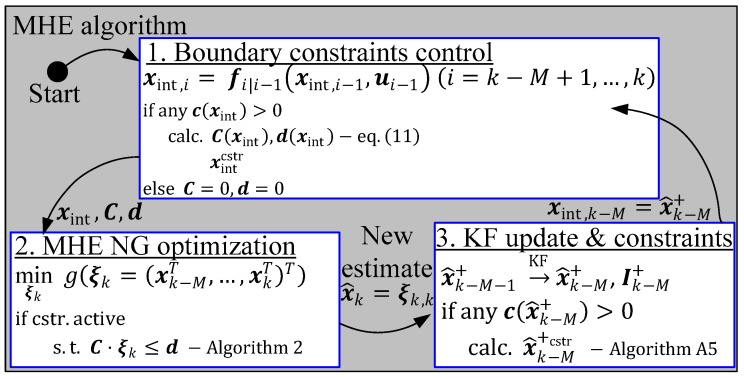
Flowchart of the extended moving horizon estimator.

With this implementation, all of the necessary quantities for the constrained MHE are nested within the multi-rate FMU block, whose interfaces to the outside (denoted with blue arrows) are the same as if no inner model separation was performed. This gives a large benefit in the matter of computational effort, not only caused by the larger integration step, but it also enables the possibility to calculate the constraint only if it is necessary for the estimation algorithm. This benefit is shown in the following simplified flow diagram of the extended MHE algorithm proposed here (see Figure 11). By means of a forward integration in step 1 from $t_{k-M}$ to $t_{k}$, it is checked whether any constraint may be potentially activated ($c^{a}$) and if so, the constraints are linearized along the open loop state trajectory. In step 2, the nonlinear gradient algorithm performs the optimization over the estimation window, incorporating the linearized constraints if necessary. In the last step, step 3, the Kalman filter is updated with the consideration of the system constraints to guarantee that the initial state of the moving window in the next iteration step lies within the feasible region.

**Figure 12:**
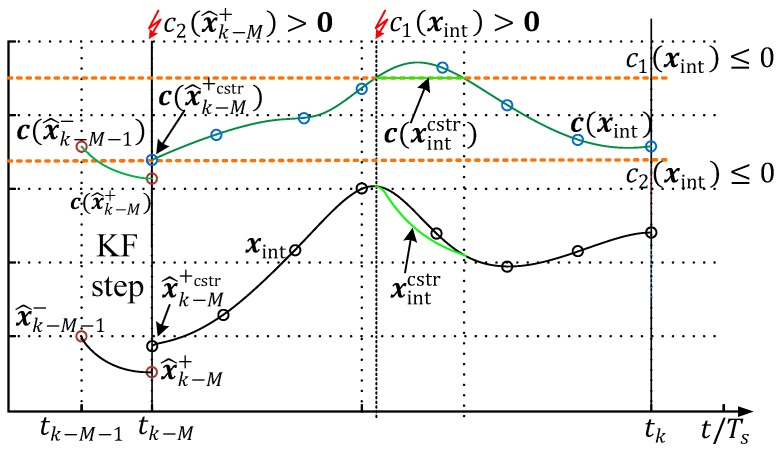
Constraint violation detection and handling within the moving window.

Qualitatively, the incorporation of the constraints is depicted in Figure 12. The feasible region of the constraint is limited through the function of $c_{1}$ and $c_{2}$ (orange-dotted). In this example, the state propagation of the Kalman filter (step 3) causes a violation of $c_{2}$, and therefore, the a posteriori propagated state must be constrained by a method (e.g., state constraint projection in Appendix B. The boundary constraints control (step 1) now starts with the corrected a posteriori estimate, ${\hat{x}}_{k-M}^{{+}_{\mathrm{cstr}}}$, and detects a constraint violation between the third and fourth sample point. This is only possible as the BC model is integrated fifty times between every ESTM evaluation and correction step. To guarantee that the initial solution of the NG solver lies in the feasible region, the $x_{\mathrm{int}}^{\mathrm{cstr}}$ is limited via the simplified Newton descent search, as described in Appendix B.

#### 3.2.2. Multiple Shooting Inspired Optimization Objective Extension

**Figure 13:**
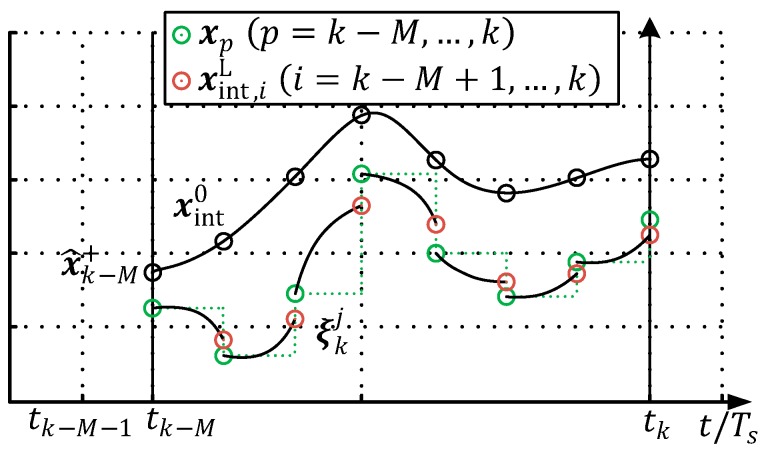
Schematic representation of a moving measurement window.

In the original MHE problem formulation (Equation (8) to Equation (10)), the discrete system states within the moving window $\xi_{k}={\left(x_{k-M}^{T},\;x_{k-M+1}^{T},\ldots ,x_{k}^{T}\right)}^{T}$ are not coupled with each other between the sample points ($t_{k}$). This implies that the optimization algorithm does not have any information about the dynamic behavior of the ESTM prediction model between the sample points within the estimation window. In the case of the ESTM, with a large sample time $T_{s}=200\;\mathrm{ms}$, this may lead to a physically unfeasible set $\xi_{k}$, which however minimizes the optimization criteria. A consideration to overcome this weak point is the introduction of coupling penalty terms between the time instances in the minimization criterion in Equations (9) and (10). Figure 13 exemplifies the approach developed here, namely: the initial open loop integration from the time instance $t_{k-M}$ to the current time instance $t_{k}$ is denoted as $x_{\mathrm{int}}^{0}$. The set of optimized state vectors $\xi_{k}^{j}$ in the j-th NG descent step (cf. Algorithm 2) is marked with green circles.

The temporal evolution from these discrete states by means of the FMU yields a set of system states $x_{\mathrm{int},i}^{L}$ denoted with a red circle, as follows:

(12) $$x_{\mathrm{int},i}^{L}=f_{i|i-1}\left(x_{i-1},u_{i-1}\right),\left(i=k-M,\ldots ,k\right)$$

In the depicted qualitative example (cf. Figure 13), one can see, that through the evolution of the optimization process a displacement $x_{\mathrm{int},i}^{L}\neq x_{p}$ is caused in the *j*-th iteration step of the NG algorithm (see Algorithm 2). To minimize this gap, the MHE optimization objective is changed to the following:

(13) $$\begin{array}{c} g\left(\xi_{k}\right)=∥x_{k-M}-{\hat{x}}_{k-M}^{+}{∥}_{I_{k-M}^{+}}^{2}\\ +\sum_{i=k-M}^{k}∥y_{i}^{m}-h\left(x_{i}\right){∥}_{R^{-1}}^{2}\\ +\sum_{i=k-M+1}^{k}∥x_{i}-x_{\mathrm{int},i}{∥}_{Q^{-1}}^{2}\\ +\underset{\mathrm{Additional}\;\mathrm{penalty}}{\underbrace{\overset{k}{\underset{i=k-M}{\sum }}∥x_{i}-x_{\mathrm{int},i}^{L}{∥}_{Q_{\mathrm{MS}}}^{2}}} \end{array}$$

In comparison to the original formulation, the quality functional is extended with an additional weighted least squares expression so as to enforce a stronger coupling of the piecewise integration ($x_{\mathrm{int},i}^{L}$) and the optimized stated vector ($x_{i}$) by means of the user tunable weighting matrix ($Q_{\mathrm{MS}}$).

Besides performing the integration ($x_{\mathrm{int},i}^{L}$) by means of the FMU (Equation (12)), it is necessary to approximate the integration rule for the gradient $g\left(\xi_{k}\right)$ calculation. It is proposed that it is more important to generate a good approximated descent direction for the optimizer than the exact reproduction of the integration method $x_{\mathrm{int},i}^{L}$ used in the FMU.

In the simplest case, this is achieved by the consideration of the directional derivative at the past instance (later called V1). In the second version (V2), the integrator is approximated as an Euler 1 integration rule. The last optimization variable coupling approximation is formulated by a trapezoid integration rule (V3), as follows:

(14) $$\begin{array}{l} V2\to x_{E1,i}^{L}=f_{i|i-1}\left(x_{i-1},u_{i-1}\right)\approx \;x_{i-1}+f_{i-1}\cdot T_{s}\\ V3\to x_{\mathrm{Tr},i}^{L}=f_{i|i-1}\left(x_{i-1},u_{i-1}\right)\approx \;x_{i-1}+\frac{1}{2}\cdot (f_{i-1}+f_{i})\cdot T_{s} \end{array}$$

With these three versions, the complete extended MHE gradient computation is given in Algorithm 4. Comparing this gradient calculation to the original formulation in Algorithm 3, the main difference is the additional “for loop” with the index j, in which the operator ± denotes that all of the values are additively added to the existing entries from the earlier loop.
**Algorithm 4.** Multiple shooting MHE gradient calculationDefine: $R^{*}={\left(R\cdot R\right)}^{-1};\;Q^{*}={\left(Q\cdot Q\right)}^{-1}$; E=eye(n)
Define: $i_{1}\left(i\right):=\;1+\left(i-k+M\right)n:\left(i-k+M+1\right)n$
$\nabla g_{1:n}=\left(I_{k-M}^{+}+{\left(I_{k-M}^{+}\right)}^{T}\right)\left(x_{k-M}-{\hat{x}}_{k-M}^{+}\right)\;-2{\frac{\partial h\left(x_{k-M}\right)}{\partial x_{k-M}}}^{T}R^{*}\left(y_{k-M}^{m}-h\left(x_{k-M}\right)\right)$**for**i=k−M+1 to k
**do**    $\nabla g_{i_{1}\left(i\right)}=\frac{\partial g\left(\xi_{k}\right)}{\partial x_{i}}=2Q^{*}\left(x_{i}-x_{\mathrm{int},i}\right)-2\frac{\partial h{\left(x_{i}\right)}^{T}}{\partial x_{i}}R^{*}\left(y_{i}^{m}-h\left(x_{i}\right)\right)$    **if** version == V3 **then**        $\nabla g_{i_{1}\left(i\right)}-={\left(\frac{1}{2}\cdot T_{s}\cdot \frac{\partial f_{i}}{\partial x_{i}}\right)}^{T}\cdot 2\cdot Q_{\mathrm{MS}}\cdot \left(x_{i}-x_{\mathrm{int},i}^{L}\right)$    **end if****end for**Define: $j_{1}\left(j\right):=1+\left(j-k+M\right)n:\left(j-k+M+1\right)n$
**for**j=k−M to k−1
**do**  $\nabla g_{j_{1}\left(j\right)}+=\frac{\partial g\left(\xi_{k}\right)}{\partial x_{j}}$  **switch**(version)    **case** V1:      $\nabla g_{j_{1}\left(j\right)}+=-\frac{\partial f_{j}}{\partial x_{j}}\cdot 2\cdot Q_{\mathrm{MS}}\cdot \left(x_{j+1}-x_{\mathrm{int},j+1}^{L}\right)$    **case** V2:      $\nabla g_{j_{1}\left(j\right)}+=-{\left(\frac{1}{2}\cdot T_{s}\cdot \frac{\partial f_{j}}{\partial x_{j}}\right)}^{T}\cdot 2\cdot Q_{\mathrm{MS}}\cdot \left(x_{j+1}-x_{\mathrm{int},j+1}^{L}\right)$
    **case** V3:      $\nabla g_{j_{1}\left(j\right)}-={\left(E+\frac{1}{2}\cdot T_{s}\cdot \frac{\partial f_{j}}{\partial x_{j}}\right)}^{T}\cdot 2\cdot Q_{\mathrm{MS}}\cdot \left(x_{j+1}-x_{\mathrm{int},j+1}^{L}\right)$  **end switch****end for**

#### 3.2.3. Multi-Rate, Triggered, or Delayed Measurements in MHE

For the nonlinear MHE approach of Section 3, it is proposed to keep the lagged sensor data in a ring buffer, to interconnect them with past measurements of non-lagged sensors, and to index the active sensors at the particular time instance. Comparing this with the formulation of Equation (9), all of the expressions that are connected with the measured output (the middle part) have to be calculated separately in each time step so as to incorporate the changing amount of available sensor information at the dedicated time step.

(15) $$\begin{array}{cc} g\left(\xi_{k}\right) & =\cdots \;+\overset{k}{\underset{i=k-M}{\sum }}∥y_{i,a}^{m}-h_{a}\left(x_{i}\right){∥}_{R_{k}^{-1}}^{2}+\cdots \\ {\bar{\sigma }}_{r_{a}} & =\;\left\{\sigma_{r_{i}}|i\in y_{k}\right\}\\ R_{k} & =\mathrm{diag}\left({\bar{\sigma }}_{r_{a}}\right)\\ h_{a} & =\left\{h_{i}|i\in y_{k}\right\}\\ y_{a}^{m} & =\left[\begin{array}{ccc} y_{k-M,1}^{m} & \ldots & y_{k,1}^{m}\\ \vdots & \ldots & \vdots \\ y_{k-M,n_{y}}^{m} & \ldots & y_{k,n_{y}}^{m} \end{array}\right] \end{array}$$

In Equation (15), all of the active measurements are denoted with an additional subscript (${\left(\cdot \right)}_{a}$) for the active sensors. In the construction of the active measurement matrix ($y_{a}^{m}$), the time-delays of the single sensors are already considered. To cope with the varying dimensions in the optimization problem, the implementation could utilize the vector indexing feature in the Modelica language. The same technique is used in the Kalman filter propagation (cf. Algorithm 1) for the reduced indexed measurements. This procedure is valid according to the Kalman theory, and can be seen in an analogy to sequential Kalman filtering (see [10] – Section 6.1). In these implementations, the matrix vector notation of the Kalman filter algorithm is replaced by sequentially solving a scalar problem for each measurement. Therefore, more sensor information can improve the estimation in the sense of minimizing the covariance, $P_{k-M}^{+}$. In the case that less sensor information is available, larger values of $\det(P_{k-M}^{+}$) occur, but the validity of the Kalman theory still holds. In the same manner, the dimension of the available measurements at $t_{k-M}$ for the calculation of the information matrix $I_{k-M}^{+}$ and the initial guess ${\hat{x}}_{k-M}^{+}$ also needs to be considered in the Kalman step.

#### 3.2.4. Adaptive Initial Reference Refreshing for Delayed Measurements

**Figure 14:**
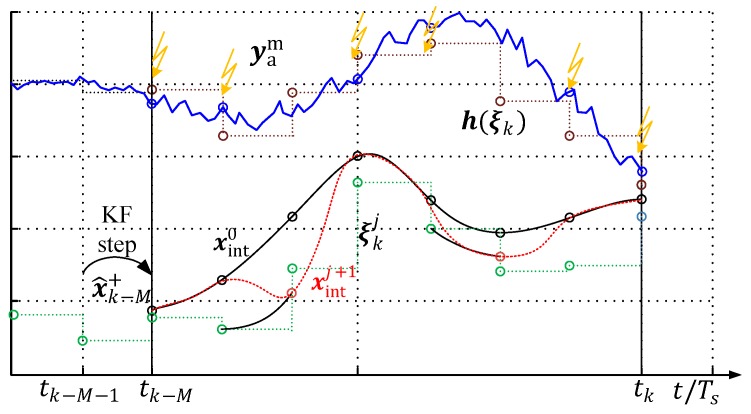
Moving horizon window with fragmentary measurements.

In Section 3.2.3, a theory extension to MHE is given that enables the assignment of measurements to their particular time instance by intelligent measurement storage and temporal activation indexing. In Figure 14, the measurement signal $y_{a}^{m}$ is schematically sketched, which is only available at the time instances highlighted with a yellow flash. For example, between time instances $t_{k-M+1}$ and $t_{k-M+3}$, no new measurement information is available.

Different algorithm experiments have shown that this may force the NG optimizer to tune the variable $\xi_{k,3}$ towards the initial guess of the open loop state trajectory ($x_{\mathrm{int}}$). To overcome this very conservative solution, a heuristic method is introduced in Equation (16) to refresh $x_{\mathrm{int}}^{j+1}$ after the optimization step (line 10 in Algorithm 2). It determines the gaps in the logical vector indexing matrix of the active measurements $y_{{\mathrm{index}}_{a}}^{m}$ and integrates from the last time instance where all of the measurements are available, as follows:

(16) $$x_{\mathrm{int},i}^{j+1}=\{\begin{array}{c} x_{\mathrm{int},i}^{0},\;\forall \;\left\{i|\left(i\in y_{a}^{m}\right)\wedge \left(i+1\in y_{a}^{m}\right)\right\}\\ f_{i|i-1}\left(\xi_{k,i-1}^{j},u_{i-1}\right),\;\forall \;\left\{i|\left(i\in y_{a}^{m}\right)\wedge \left(i+1\notin y_{a}^{m}\right)\right\},\;\left(i=k-M,\ldots ,k\right)\\ f_{i|i-1}\left(x_{\mathrm{int},i-1}^{j+1},u_{i-1}\right),\forall \;\left\{i|\left(i\notin y_{a}^{m}\right)\wedge \left(i+1\notin y_{a}^{m}\right)\right\} \end{array}$$

## 4. Experimental Results

**Figure 15:**
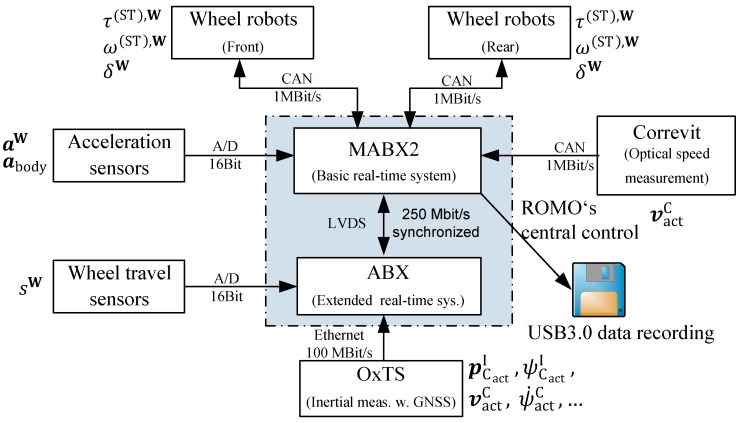
ROboMObil’s vehicle dynamics sensors and actuators architecture. OxTS—inertial measurement unit; ABX—AutoBox (rapid prototyping real-time controller); MABX2—MicroAutoBox II (embedded rapid prototyping real-time controller).

The following experimental results rely on the test measurement data that were recorded during the driving experiments using ROboMObil, as described in Section 2. Two real-time rapid prototyping systems were used to record all of the variables from the wheel robot controllers as well as the senor system of ROboMObil. The arrangement, shown in Figure 15, is designed to meet the robotics inspired central control architecture with two synchronized rapid prototyping controllers (dSPACE MABX2 and ABX), which represent ROboMObil’s central control unit. The wheel robots with the traction and steering drive torques ($\tau^{\left(\mathrm{ST}\right),W}$), angular velocities ($\omega^{\left(\mathrm{ST}\right),W}$), and steering angles ($\delta^{W}$) quantities, as well as the Correvit optical measurement unit (used to measure the vehicle’s longitudinal and lateral speed $v_{\mathrm{act}}^{C}=\left\{v_{{\mathrm{act}}_{x}}^{C},v_{{\mathrm{act}}_{y}}^{C}\right\}$), are connected via high-speed controller area network (CAN) busses. The inertial measurement system with a differential corrected Global Navigation Satellite System (GNSS) to measure the vehicle states, such as the positon $p_{C_{\mathrm{act}}}^{I}$, yaw angle $\psi_{C_{\mathrm{act}}}^{I}$ and yaw rate ${\dot{\psi }}_{C_{\mathrm{act}}}^{I}$, or the velocity $v_{\mathrm{act}}^{C}$, is wired to the central control by an Ethernet bus. Additionally, the wheel ($a^{W}$) and chassis body ($a_{\mathrm{body}}$) accelerations, as well as wheel travel sensors ($s^{W}$) are tethered via analog inputs. Both the inertial measurement unit from Oxford Technical Solutions Ltd. (OxTS) measurement unit and the optical odometry sensor are suited for the experimental evaluation of the driving experiments.

### 4.1. Observer Configuration

The preliminary observer case studies showed that with the modeling approach chosen here (cf. Section 2.1), in combination with a Runge–Kutta 4 integrator, the vehicle position estimator can be run with a cycle time of $T_{s}=200\;\mathrm{ms}$. Moreover, the usage of the vehicle yaw rate ($\;{\dot{\psi }}^{C}$) can actively contribute to improve the measurement, whereas the incorporation of the vehicle lateral acceleration ($a_{y}^{C}$) downgrades the observer performance. The output equation, $a_{y}^{C}=v^{C}\;\cdot \;\left({\dot{\psi }}^{C}+{\dot{\beta }}^{C}\right)$, shows clearly that the lateral acceleration is algebraically cross-coupled to the vehicle yaw rate (${\dot{\psi }}^{C}$). In fact, this causes the aforementioned observations, as it is, to the best knowledge of the author, impossible to find a covariance configuration—even by optimization—which makes reasonable use of both the sensor information and overcomes the negative effects of, for example, minimal jittering relative delays between both signals.

**Figure 16:**
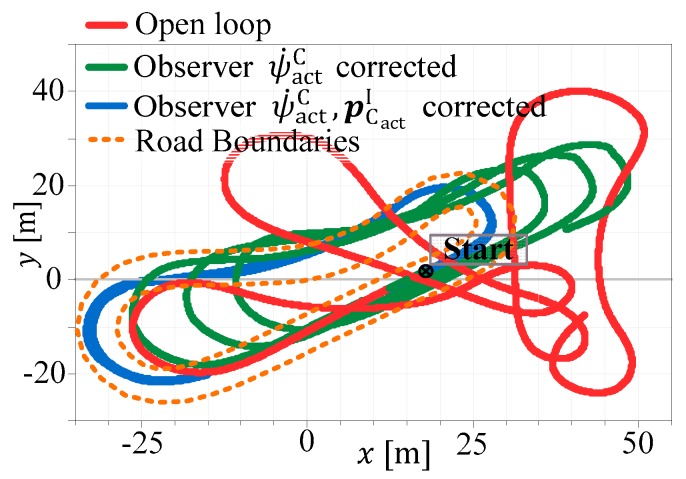
Comparison of different measurement incorporations.

In Figure 16, different simulation results of the same vehicle position estimator are given. Again, the road boundaries are marked in a dotted-orange color. The vehicle completed three rounds through the circuit. The red trajectory denotes the open loop result—the ESTM model is simulated with the actual system inputs u—and the position is strongly drifting away from the planned trajectory, caused by the evolving position integration error (compare equations for $dx_{C}/dt,\;dy_{C}/dt$—in Equation (A1)). The green and the blue curves denote the results with a single step Kalman filter, under the assumption that no delays are in the measurements.

The best observation results could be achieved by the use of a square root extended Kalman filter (SR-EKF) algorithm (cf. e.g., [2]), whereas the square root unscented Kalman filter (SR-UKF) (cf. e.g., [2]) was less robust. The reason for this is the sigma point propagation through the Pacejka tire model, which evidently leads to a wrong state propagation caused by its high sensitivity around the actual state estimation point.

### 4.2. Moving Horizon Estimation Algorithm Assessment

In this last subSection, the complete MHE ESTM algorithm with its extensions to couple the optimization variables (cf. Section 3.2), refreshment of the initial guess, and efficient road boundary constraint handling are demonstrated (cf. Figure 7). In Table 1, the outcome of a comprehensive simulation case study is summarized. The window length has been set to M=4 and the GPS time delay varies between $n_{d}=0\ldots 3$ steps. All of the parameters for the respective observer configuration have been optimized with the DLR multi-objective parameter synthesis (MOPS) optimizer framework [27] to guarantee comparable results.

The results in Table 1 are sorted with respect to the number of delayed samples ($n_{d}$) of the GPS signal. The results that differ tremendously (good or bad) in a group of the same number of delays are highlighted in green or red, respectively. The first column denotes the used observer setup, in which the acronyms for SS (single shooting; standard formulation), MS (multiple shooting), fix (constant initial guess), and up (anti optimization freezing) are used. The second column gives the average execution time per estimation step ${\bar{T}}_{\mathrm{mean}}$ in [ms] of the experiment executed on a standard 64-bit Windows-based system (Intel i7-4600U 2,7 GHz, 8 GB Ram, SSD), and can be interpreted as a measure for the increase of computational complexity in comparison to the improvement of the estimation. It can be seen that even in the most computationally demanding configuration (MHE MS up V3 ${\bar{T}}_{\mathrm{mean}}=136.8\;\mathrm{ms}$), the algorithm can be executed faster than in real-time (${\bar{T}}_{\mathrm{mean}}\ll T_{s}$). In the third to eighth columns, the percentage goodness of fit (normalized root mean square error between to time series) in comparison to the reference measures of the experiment (cf. Section 2) are summarized.

The first two rows in Table 1 give reference of the ESTM without any observer correction, but in the second row has the roadway constraint incorporation. The next two rows are the first results using an observer that only incorporates the measured vehicle yaw rate (${\dot{\psi }}_{\mathrm{act}}^{C}$). Both still have a large positional deviation, albeit the estimation of the vehicle yaw rate, and the angle and side slip angle are improved in comparison with the open loop tests. The next section highlights three versions of the ESTM observer, with all measurements ${\dot{\psi }}_{\mathrm{act}}^{C},\;p_{C_{\mathrm{act}}}^{I}$ available and not delayed. The best results can be achieved with the multiple shooting objective V1. The following two experiments incorporate a delay of one sample step.

**Figure 17:**
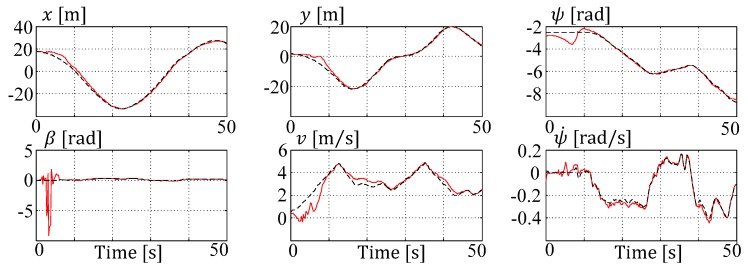
Extended single track model (ESTM) square-root extended Kalman filter (SR-EKF) ($n_{d}=1$) setup state estimations (red) vs. reference (black).

Unfortunately, the SR-EKF algorithm could not be stabilized to give a feasible estimate for all of the measures, especially the side slip angle ($\beta^{C}$) is heavily oscillating (cf. Figure 17—bottom left). Here, for the first time, the delayed measurement compensation in the MHE formulation can show its advantage in the single shooting as well as in the multiple shooting objective formulations.

**Figure 18:**
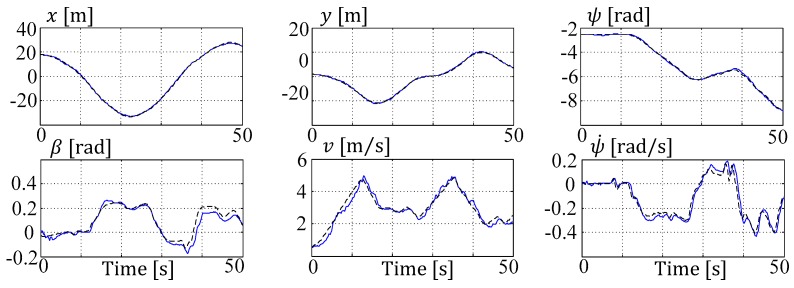
ESTM moving horizon estimation/estimator multiple shooting (MHE MS) up V1 ($n_{d}=2$) state estimations (blue) vs. reference (black).

The section with $n_{d}=2$ is the largest section, which correlates to a delay of 400 ms. All of the modifications introduced in Section 3.2 are tested here for their performance. Even though all of the results are very close to each other, the multiple shooting V1 with reference updating gives the best performance in positioning accuracy and computational reliability (cf. state plots in Figure 18).

**Figure 19:**
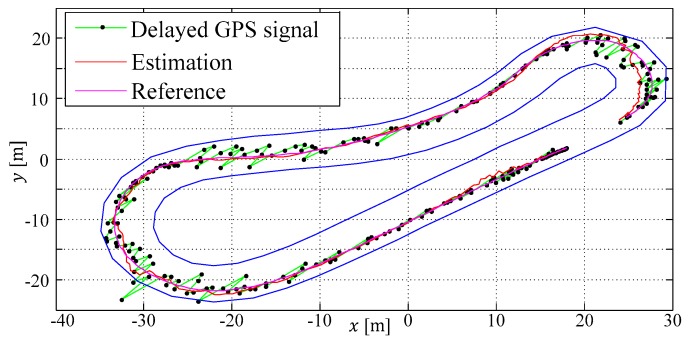
ESTM MHE single shooting (SS) up ($n_{d}=3$): x–y position estimations results.

In the last two rows of the simulation study, two configurations with $n_{d}=3$ are assessed. The single shooting (see Figure 19) and multiple shooting do benefit from the proposed update mechanism, and even if the GPS signal is delayed by 600 ms, the results are still reliable.

**Figure 20:**
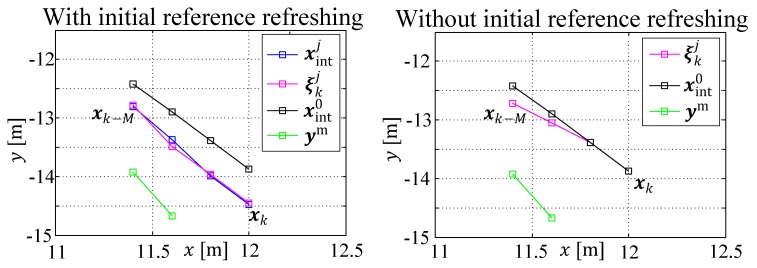
Effectiveness of the anti-freezing heuristic within the optimization window.

In total, it can be stated that the time delay incorporation in a MHE is very effective and gives good stability, especially when the sample steps are large. The competitive SR-EKF algorithm failed already with a delay of $n_{d}=1$, although its computational time is up to 20 times lower. The influence of the extension of the objective function with multiple shooting penalties needs to be analyzed from case to case, whereas the anti-freezing feature in the optimizer can be seen as a good improvement to the solution at time instance $t_{k}$ (cf. Figure 20).

Future investigations will be performed with methods other than the NG solver methods, as recently proposed in the literature [28], which are capable of handling nonlinear (in-)equality constraints in real-time. With this extension, it is likely that the multiple shooting approaches might be even more effective.

## 5. Conclusions

**Figure 21:**
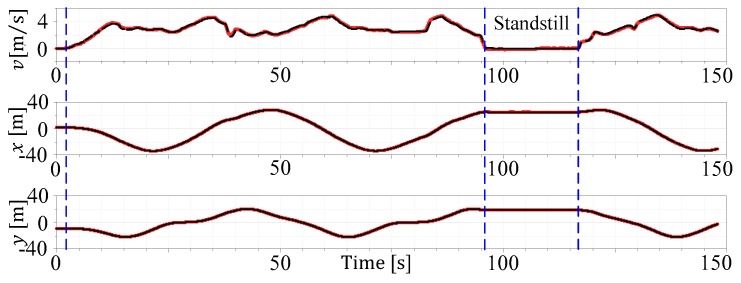
Experiment with vehicle standstill estimation (red) vs. reference (black).

The outcome of the ESTM MHE observer can be summarized as follows:

- A continuous-time Modelica vehicle model with event handling for vehicle standstill (cf. Section 2.1) could be derived (see estimation experiment in Figure 21, starting from standstill and coming back to standstill for about 20 s) and automatically discretized via Dymola, the extended FMU 2.0 for co-simulation technology and the model-based observer framework.
- The derivation of the roadway limit constraint has been designed by extending the principle of the path interpolation introduced in Section 2.2.
- The nominal MHE algorithm of Section 3 was augmented with a multiple shooting formulation in the objective function, a heuristic optimization freezing prevention, a multi-rate model, and a constraint calculation splitting methodology.
- For the experimental investigations, real test data from ROMO was selected, and additionally, the GPS position measures were delayed for the experimental setup.
- All together with the technique for delayed measurements in the MHE application (Section 3.2.3), a comprehensive simulative assessment with the different objective configuration and anti-freezing features, as well as varying delays in comparison to a standard Kalman filter, have been given.
- The proposed estimation approach could achieve a position $x_{C},y_{C}$ estimate fit of about 98%, and by mean of the delay compensation technique, this value is, even with a delay of $n_{d}=3$, only reduced to about 97%. The estimate of the vehicle velocity is even 4% improved by the use of the MHE technique in comparison to the EKF. The yaw angle estimate ($\psi_{C}$) quality for all of the configurations is very high, whereas the yaw rate (${\dot{\psi }}^{C}$) and side slip angle ($\beta^{C}$) do vary about 5%, but still have reasonable estimates.

## Figures and Tables

**Table 1:** Comparison of the vehicle position observer performance indicators. SR-EKF—square-root extended Kalman filter; MHE—moving horizon estimation/estimator; MS—multiple shooting; SS—single shooting.

Algorithm	${\bar{T}}_{\mathrm{mean}}$	$x_{C_{\mathrm{Fit}}}$	$y_{C_{\mathrm{Fit}}}$	$\psi_{C_{\mathrm{Fit}}}$	${\dot{\psi }}_{\mathrm{Fit}}^{C}$	$v_{\mathrm{Fit}}^{C}$	$\beta_{\mathrm{Fit}}^{C}$
$n_{d}=-$							
Open Loop	6.4	75.5	62.2	96.4	80.2	77.7	81.0
Open Loop Cstr.	6.4	87.7	87.5	96.4	80.2	77.7	81.0
$n_{d}=0$							
$\mathrm{SR}\text{-}\mathrm{EKF}\;{\dot{\psi }}_{\mathrm{act}}^{C}$	4.4	78.9	95.2	99.1	95.5	77.3	87.4
$\mathrm{SR}\text{-}\mathrm{EKF}\;{\dot{\psi }}_{\mathrm{act}}^{C}$ Cstr.	4.8	88.2	89.2	99.1	95.5	77.3	87.4
SR-EKF	6.4	97.9	97.2	99.2	93.3	82.4	85.2
MHE SS	67.2	97.3	96.5	99.4	95.5	83.1	81.3
MHE MS V1	46.4	98.5	97.8	99.3	86.9	86.4	80.4
$n_{d}=1$							
MHE SS up	78.8	98.2	97.8	99.4	98.4	84.6	80.9
MHE MS up V1	93.6	98.3	97.8	99.4	86.8	85.3	81.0
$n_{d}=2$							
MHE SS fix	77.6	98.4	97.8	99.2	98.9	85.9	80.1
MHE SS up	75.2	98.7	98.1	99.2	98.2	85.9	80.3
MHE MS up V1	61.6	98.8	98.2	99.3	86.9	86.4	80.4
MHE MS fix V2	64.8	98.5	98.1	99.2	86.3	86.7	80.0
MHE MS up V2	110.0	98.7	98.2	99.2	86.8	86.7	80.0
MHE MS fix V3	67.2	98.5	98.0	99.2	86.5	86.6	80.3
MHE MS up V3	136.8	98.7	98.3	99.2	86.6	86.6	80.3
$n_{d}=3$							
MHE SS up	56.8	97.7	97.1	98.9	97.3	86.8	79.6
MHE MS up V1	79.6	97.7	97.1	99.1	87.9	87.3	80.1

**Figure A1:**
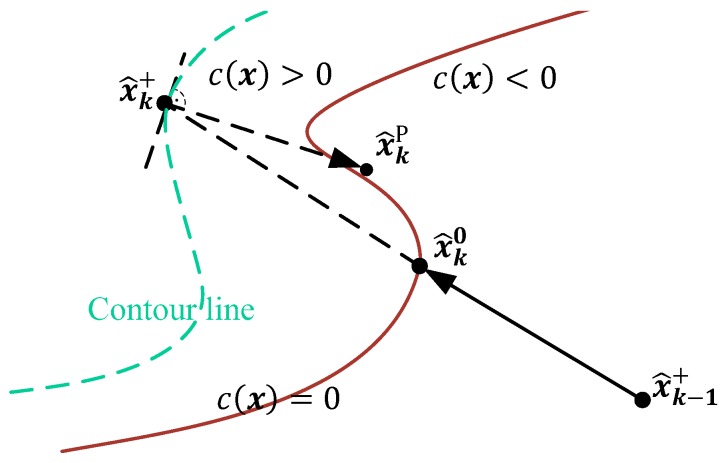
Principle of the Newton descent search for state constraint handling.

## Appendix A. The Extended Nonlinear Single Track Model Equations

The set of ordinary differential equations of the ordinary differential equation form $x^{C}=\left\{\beta^{C},v^{C},{\dot{\psi }}^{C},\;\psi_{C},x_{C},y_{C}\right\}$ can be given as follows:

(A1) dβCdt=−sin(βC)FxC+cos(βC)FyCm⋅vmodC−ψ˙CdvCdt=cos(βC)FxC+sin(βC)FyCmdψ˙Cdt=MZC/JZCdψCdt=ψ˙CdxCdt=vC⋅cos(ψC+βC)dyCdt=vC⋅sin(ψC+βC)

The vehicle side slip ($\beta^{C}$) is the angle between the vehicle origin coordinate system and the actual vehicle velocity vector ($v^{C}$). The yaw angle ($\psi_{C}$) and yaw angle rate (${\dot{\psi }}^{C}$) describe the rotation with respect to a fixed inertial system ($x^{I},y^{I}$), in which the vehicle positions $x_{C},y_{C}$ are also expressed. Additional quantities are the vehicle mass (m), the yaw moment around the center of gravity ($M_{Z}^{C}$), and the corresponding vehicle yaw inertia ($J_{Z}^{C}$). To prevent division by zero, the denominator of the vehicle side slip state (${\dot{\beta }}^{C}$) is calculated by use of Equation (A2), which limits the minimum velocity to $v_{\min}^{C}$ (only valid for small values of $v_{\min}^{C}$).

(A2) $$v_{\mathrm{mod}}^{C}=\frac{\sqrt{v^{C}\cdot v^{C}+4\cdot v_{\min}^{C}\cdot v_{\min}^{C}}+v^{C}}{2}$$

The inputs to the model are the commanded torque set-points to the in-wheel motors and the per axle averaged steering angles $u^{C}=\left\{\tau^{W_{1}},\tau^{W_{2}},\tau^{W_{3}},\tau^{W_{4}},\delta^{W_{f}},\delta^{W_{r}}\right\}$. The forces (marked blue in Figure 3) to the vehicle body center of gravity are calculated by considering the geometric dependencies to the instantaneous steering angles $\delta^{\left(\cdot \right)}$, as follows:

(A3) $$\begin{array}{cc} F_{x}^{C} & =-\sin\left(\delta^{W_{f}}\right)F_{s}^{W_{f}}\;-\sin\left(\delta^{W_{r}}\right)F_{s}^{W_{r}}+\cos\left(\delta^{W_{f}}\right)F_{l}^{W_{f}}+\cos\left(\delta^{W_{r}}\right)F_{l}^{W_{r}}-F_{{\mathrm{Air}}_{x}}^{C}\\ F_{y}^{C} & =\cos\left(\delta^{W_{f}}\right)F_{s}^{W_{f}}+\cos\left(\delta^{W_{r}}\right)F_{s}^{W_{r}}+\sin\left(\delta^{W_{f}}\right)F_{l}^{W_{f}}+\sin\left(\delta^{W_{r}}\right)F_{l}^{W_{r}}-F_{{\mathrm{Air}}_{y}}^{C}\\ M_{Z}^{C} & =l_{f}\cdot \cos\left(\delta^{W_{f}}\right)F_{s}^{W_{f}}\\ & -l_{r}\cdot \cos\left(\delta^{W_{r}}\right)F_{s}^{W_{r}}+l_{f}\cdot \sin\left(\delta^{W_{f}}\right)F_{l}^{W_{f}}\\ & -l_{r}\cdot \sin\left(\delta^{W_{r}}\right)F_{l}^{W_{r}}+e_{\mathrm{CoG}}\cdot F_{{\mathrm{Air}}_{y}}^{C}\\ F_{{\mathrm{Air}}_{y}}^{C} & =0.5\cdot c_{w_{y}}\cdot \rho \cdot A_{y}\cdot v_{\mathrm{Air}}^{C}{{}_{y}}^{2} \end{array}$$

In this set of equations, $F_{s}^{\left(\cdot \right)}$ denotes the wheel side forces and $F_{l}^{\left(\cdot \right)}$ the corresponding longitudinal forces. The distances from the center of gravity (CoG) to the rear and front wheels are given by $l_{\left(\cdot \right)}$, and the external longitudinal $F_{{\mathrm{Air}}_{x}}^{C}$ and lateral $F_{{\mathrm{Air}}_{y}}^{C}$ air drag forces are applied to the vehicle body. The lateral forces of the tire are calculated by trigonometric functions based on Pacejka’s tire model [18], whose input variable is the wheel’s side slip angle $\alpha^{W_{\left(\cdot \right)}}$, as follows:

(A4) $$\begin{array}{l} F_{s}^{W_{f}}=D\cdot \sin\left(C\cdot \mathrm{atan}\left(B\cdot \alpha^{W_{f}}-E\cdot \left(B\cdot \alpha^{W_{f}}-\mathrm{atan}\left(B\cdot \alpha^{W_{f}}\right)\right)\right)\right)\\ F_{s}^{W_{r}}=D\cdot \sin\left(C\cdot \mathrm{atan}\left(B\cdot \alpha^{W_{r}}-E\cdot \left(B\cdot \alpha^{W_{r}}-\mathrm{atan}\left(B\cdot \alpha^{W_{r}}\right)\right)\right)\right)\\ \alpha^{W_{f}}=\left(\delta^{W_{f}}\right)-\mathrm{atan}\left(\frac{v_{\mathrm{mod}}^{C}\cdot \sin\left(\beta^{C}\right)+l_{f}\cdot {\dot{\psi }}^{C}}{v_{\mathrm{mod}}^{C}\cdot \cos\left(\beta^{C}\right)}\right)\\ \alpha^{W_{r}}=\left(\delta^{W_{r}}\right)-\mathrm{atan}\left(\frac{v_{\mathrm{mod}}^{C}\cdot \sin\left(\beta^{C}\right)-l_{r}\cdot {\dot{\psi }}^{C}}{v_{\mathrm{mod}}^{C}\cdot \cos\left(\beta^{C}\right)}\right) \end{array}$$

Herein, the factor B denotes the tire’s stiffness, C is an aspect ratio for the calculated force, D the maximum value of the curve, and E is the modulus of bending rupture. For the longitudinal tire dynamics, a simplified modeling without the consideration of the actual wheel slip was used, as experiments in combination with observer algorithms yielded poor and unstable results, and the vehicle’s acceleration range of the maneuvers considered here is limited, as follows:

(A5) $$\begin{array}{l} F_{L}^{W_{f}}=\frac{\tau^{W_{1}}}{R}+\frac{\tau^{W_{2}}}{R}-f_{\mathrm{rv}}\cdot \left(\frac{m\cdot l_{r}\cdot g}{l_{f}+l_{r}}\right)\\ F_{L}^{W_{r}}=\frac{\tau^{W_{3}}}{R}+\frac{\tau^{W_{4}}}{R}-f_{\mathrm{rv}}\cdot \left(\frac{m\cdot l_{f}\cdot g}{l_{f}+l_{r}}\right) \end{array}$$

Finally, the speed dependent rolling resistance ($f_{\mathrm{rv}}$( and the longitudinal air drag ($F_{{\mathrm{Air}}_{x}}^{C}$) are calculated according to Equation (A6). By use of the “if-statement”, the vehicle is prevented from rolling backwards in case of standstill on a planar surface, and no braking torque is applied, as follows:

(A6) if vC>vminC  frv=fR0+fR1⋅vmodC100+fR4⋅(vmodC100)4  FAirxC=12⋅cwx⋅ρ⋅Ax⋅vAirCx2else  frv=0 FAirxC=0end

In the following the result of the STM parameter, identification by means of a nonlinear model based optimization is given (cf. Table A1). The derived model is implemented in Modelica and later exported as an FMU for the observer, described in Section 3.2. For the optimization process, the DLR Optimization Library [29] has been utilized. As a tuning algorithm, the pattern search method was chosen. For training and validation, data real world experiments on vehicle test tracks with ROboMObil have been used. ROMO’S ESTM optimized parameters are given in Table A1.

**Table A1:** ROMO’s ROboMObil’s—(German Aerospace Center’s (DLR’s) robotic electric vehicle) extended single track model (ESTM) optimized parameters.

Parameter	Value	Description
$f_{R0}$	0.009	First parameter of roll resistance
$f_{R1}$	0.2811588	Second parameter of roll resistance
$f_{R4}$	0.44906	Fourth parameter of roll resistance
B	5.1088547	Parameter of Pacjeka’s MF
C	2.0280	Parameter of Pacjeka’s MF
D	724.70	Parameter of Pacjeka’s MF
E	0.8903703	Parameter of Pacjeka’s MF
$c_{w_{x}}$	0.3	Longitudinal air drag coefficient
ρ	$1.249512\;\left[N/m^{2}\right]$	Air density
$A_{x}$	$1.2323485\;\left[m^{2}\right]$	Effective flow surface front
m	1013 [kg]	ROboMObil mass
$l_{f}$	1.218 [m]	CoG to front wheel
$l_{r}$	1.182 [m]	CoG to rear wheel
R	0.3722 [m]	Wheel radius
$J_{Z}^{C}$	$1130\;\left[{\mathrm{kgm}}^{2}\right]$	Chassis moment of yaw inertia

## Appendix B. A Constraint Projection Method for Kalman Filters

In the literature [2], a survey on different methods for incorporating inequality constraints to Kalman filter algorithms is given, and three methods for nonlinear Kalman filters that fit for constraints of the type c(x)≤0, which are neither contradictory nor redundant, and only one constraint is active at the same time, are discussed. The algorithm applied here is based on a simplified Newton descent search (i.e., without considering the second order derivative), which projects the a posteriori estimation (${\hat{x}}_{k}^{+}$) of a Kalman filter on the constrained surface. The optimization objective is formulated as follows:

(A7) $$\begin{array}{c} {\hat{x}}_{k}^{P}=\underset{x}{\mathrm{argmin}}\;\|x-{\hat{x}}_{k}^{+}\|\\ s.t.c\left(x\right)<=0 \end{array}$$

The idea behind this algorithm is to perform a descent search along the gradient ($\nabla c\left({\hat{x}}_{k}^{+}\right)$) calculated at the point ${\hat{x}}_{k}^{+}$, until the constraint equation $c\left({\hat{x}}_{k}^{P}\right)\leq 0$ holds again. Graphically this can be interpreted as shown in Figure A1.

This optimization task can be formulated with the method of Lagrange multipliers. Equation (A8) denotes the above formulated optimization objective in a general description, as follows:

(A8) minℒ(x,μ)=f(x)−μ⋅(g(x)−d)

Therefore, the optimal solution of this unrestricted minimization problem is formulated as follows:

(A9) $$\nabla_{x,\mu }ℒ\left(x,\mu \right)=0$$

This approach is now applied to the estimation projection optimization problem (Equation (A7)) and the searched restricted estimate (${\hat{x}}_{k}^{P}$) is calculated using the Lagrange multiplier (μ) and the gradient of the active constraint (c(x)), as follows:

(A10) $$\begin{array}{cc} {\hat{x}}_{k}^{P} & ={\hat{x}}_{k}^{+}+\mu \cdot \nabla c\left(x\right)\;s.t.c\left(x\right)=0\\ c\left({\hat{x}}_{k}^{+}+\mu \cdot \nabla c\left(x\right)\right) & =0 \end{array}$$

To fulfill Equation (A10), the approach is expressed with a simplified Newton descent search algorithm by means of a scalar zero search F(μ)=0, with respect to the Lagrange variable μ, as follows:

(A11) $$\begin{array}{cc} \nabla c\left(x\right) & \approx \nabla c\left({\hat{x}}_{k}^{+}\right)\\ \overset{\mathrm{yields}}{\to }F\left(\mu \right) & =c\left({\hat{x}}_{k}^{+}+\mu \cdot \nabla c\left({\hat{x}}_{k}^{+}\right)\right)\overset{!}{=}0\\ \frac{\partial F}{\partial \mu } & =\nabla c{\left({\hat{x}}_{k}^{+}+\mu \cdot \nabla c\right)}^{T}\cdot \nabla c\left({\hat{x}}_{k}^{+}\right)\\ {\frac{\partial F}{\partial \mu }|}_{\mu =0} & =\nabla c{\left({\hat{x}}_{k}^{+}\right)}^{T}\nabla c\left({\hat{x}}_{k}^{+}\right) \end{array}$$

This can be implemented as an iterative search algorithm, which determines μ such that the condition F(μ)=0 holds within a predefined maximum number of calculation steps. The pseudo code for the algorithm is given in Algorithm A1.

**Algorithm A1.** Simplified Newton descent search algorithm for constrained estimation projection.
**1.** Set $\mu_{o}=0$ and k=−1 and iter=1 and max_iter=30
Set $\alpha ={\left(\nabla c{\left({\hat{x}}_{k}^{+}\right)}^{T}\cdot \nabla c\left({\hat{x}}_{k}^{+}\right)\right)}^{-1}$
**while**$\left|\alpha \cdot c\left({\hat{x}}_{k}^{+}+\mu_{k+1}\cdot \nabla c\left({\hat{x}}_{k}^{+}\right)\right)\right|\geq \epsilon_{\mathrm{Newton}}$*and iter < max_iter **do***
k=k+1
$\Delta \mu_{k+1}=-\alpha \cdot c\left({\hat{x}}_{k}^{+}+\mu_{k}\cdot \nabla c\left({\hat{x}}_{k}^{+}\right)\right)$
$\mu_{k+1}=\mu_{k}+\Delta \mu_{k+1}$
iter=iter+1
**end while**
*Solution:*${\hat{x}}_{k}^{P}={\hat{x}}_{k}^{+}+\mu_{k+1}\cdot \nabla c\left({\hat{x}}_{k}^{+}\right)$

## Appendix C. Lists of Symbols, Nomenclatures, and Abbreviations

**Formula symbol**	**Unit**	**Description**
$\beta^{C}$	[rad]	Vehicle’s side slip angle
$v^{C}$	[m/s]	Vehicle’s velocity over ground
${\dot{\psi }}^{C}$	[rad/s]	Vehicle’s yaw rate
$\psi_{C}$	[rad]	Vehicle’s yaw angle
$x_{C}$	[m]	Vehicle’s position in x direction
$y_{C}$	[m]	Vehicle’s position in y direction

**Nomenclature**	**Explanation**
e	Lower case letter variable is a scalar
e	Bold lower case letter variable is a vector
E	Bold upper case letter variable is a matrix
$e=\left\{e_{1},e_{2}\right\}$ $={\left(e_{1},e_{2}\right)}^{T}$	Equivalent vector notations
$E=\left[\begin{array}{cc} e_{11} & e_{12}\\ e_{21} & e_{22} \end{array}\right]$	Matrix notation
${\left(\cdot \right)}^{W_{x}}$	Quantity expressed in the x-th wheel robot coordinate system parallel to the car coordinate system
${\left(\cdot \right)}^{C}$	Quantity expressed in the car coordinate system with origin in CoG
${\left(\cdot \right)}_{C}^{P}$	Car quantity expressed in the path coordinate system with origin in CoG
${\left(\cdot \right)}_{C}$	Car quantity expressed in the inertial coordinate system – short for ${\left(\cdot \right)}_{C}^{I}$
${\left(\cdot \right)}_{P}$	Quantity expressed in the path coordinate system
$\underset{x}{\min}\|e{\left(x\right)\|}_{W}$ s.t.	With W weighted least squares minimization of function e with respect to x“subject to” if the problem is restricted

**Abbreviation**	**Explanation**
ABX	AutoBox—rapid prototyping real-time controller from dSPACE GmbH
ACADO	Software environment and algorithm collection for automatic control and dynamic optimization [20]
ADAC	General German Automobile Club
BC	Boundary constraint
CAN	Controller area network—vehicle bus standard
CoG	Center of gravity
DLR	German Aerospace Center
Dymola	Modelica simulator from Dassault Systèmes
EKF	Extended Kalman filter
ESTM	Extended single track model
FIFO	First in first out (buffer)
FMI	Functional mockup interface
FMU	Functional mockup unit
GNSS	Global Navigation Satellite System
GPS	Global Positioning System
IMU	Inertial measurement unit
INS	Inertial navigation system
LAPACK	Linear algebra package
MABX2	MicroAutoBox II—embedded rapid prototyping real-time controller from dSPACE GmbH
MHE	Moving horizon estimation/estimator
Modelica	Object oriented modeling language for multiphysical systems
NG	Nonlinear gradient (descent search)
OxTS	Inertial measurement unit from Oxford Technical Solutions Ltd.
PFC	Path following control
QP	Quadratic program
ROboMObil	see ROMO
ROMO	short for ROboMObil—DLR’s robotic electric vehicle
RTI	Real-time iteration
SPKF	Sigma point Kalman filter
SQP	Sequential quadratic program
SR	Square-root
SR-EKF	Square-root extended Kalman filter
SR-UKF	Square-root unscented Kalman filter
TIPI	Time independent path interpolation
TM	Traction motor
UKF	Unscented Kalman filter
Wheel robot	Wheel unit that integrates propulsion, steering, and brake c.f. [1]
